# Florid Cystic Endosalpingiosis (Müllerianosis) in Pregnancy

**DOI:** 10.1155/2016/8621570

**Published:** 2016-09-07

**Authors:** José Morales-Roselló, Loida Pamplona-Bueno, Beatriz Montero-Balaguer, Domingo Desantes-Real, Alfredo Perales-Marín

**Affiliations:** ^1^Servicio de Obstetricia, Hospital Universitario y Politécnico La Fe, Valencia, Spain; ^2^Servicio de Anatomía Patológica, Hospital Universitario y Politécnico La Fe, Valencia, Spain

## Abstract

Cystic endosalpingiosis refers to the existence of heterotopic cystic müllerian tissue resembling structures of the fallopian tubes. We report a case of florid cystic endosalpingiosis discovered in a pregnant woman during a scheduled cesarean section and review the current knowledge of this disease. A 30-year-old woman with a twin pregnancy attended the hospital day unit at term. The first twin was in a breech presentation and a cesarean section was scheduled. During the procedure the uterine fundus and part of the body were seen completely seeded with multitude of cyst-like structures resembling hydatids of Morgagni. The immunohistochemistry analysis showed a positive expression for PAX8 (Box-8), CK7, and estrogen and progesterone receptors. The lesions did not disappear after pregnancy. Cystic endosalpingiosis should be always borne in mind, even in pregnancy, when it comes to making the differential diagnosis of a pelvic or systemic multicystic mass.

## 1. Introduction

Cystic endosalpingiosis is a rare disorder caused by the heterotopical presence of tissue resembling structures of the fallopian tubes [1]. It can be considered part of a wider group of anomalies of embryological origin called müllerianosis [2] consisting in the heterotopic presence of müllerian-derived tissue in pelvic organs, or in distant locations. Although müllerian-derived tissues are sensitive to estrogen and progesterone, reports of cystic endosalpingiosis and other forms of müllerianosis in pregnancy are very scarce. We report a case of florid cystic endosalpingiosis discovered in a pregnant woman during a scheduled cesarean section and review the current knowledge of this disease.

## 2. Case Presentation

**Figure 1:**
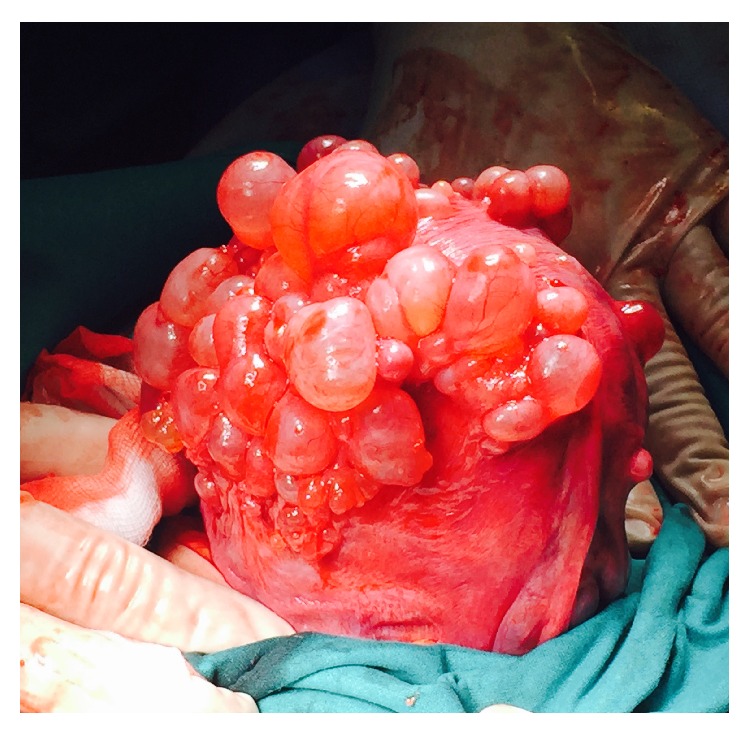
Macroscopic view of the cystic endosalpingiosis lesions after the cesarean delivery. The uterine fundus and part of the body are completely seeded with multitude of cyst-like structures resembling hydatids of Morgagni but with a harder structure due to the muscular component.

A 30-year-old woman with no remarkable past medical history and an uneventful follow-up of a bichorial-biamniotic twin pregnancy attended the hospital day unit at term for fetal growth surveillance and heart rate monitoring. The first twin was in a breech presentation and a cesarean section was scheduled at 39 weeks. During the procedure and after the extraction of both placentas, the uterine fundus and part of the body were seen completely seeded with multitude of cyst-like structures resembling hydatids of Morgagni but with a harder consistency (Figure 1). A sample of the cysts fluid and a couple of entire cysts were sent for anatomopathological study.

**Figure 2:**
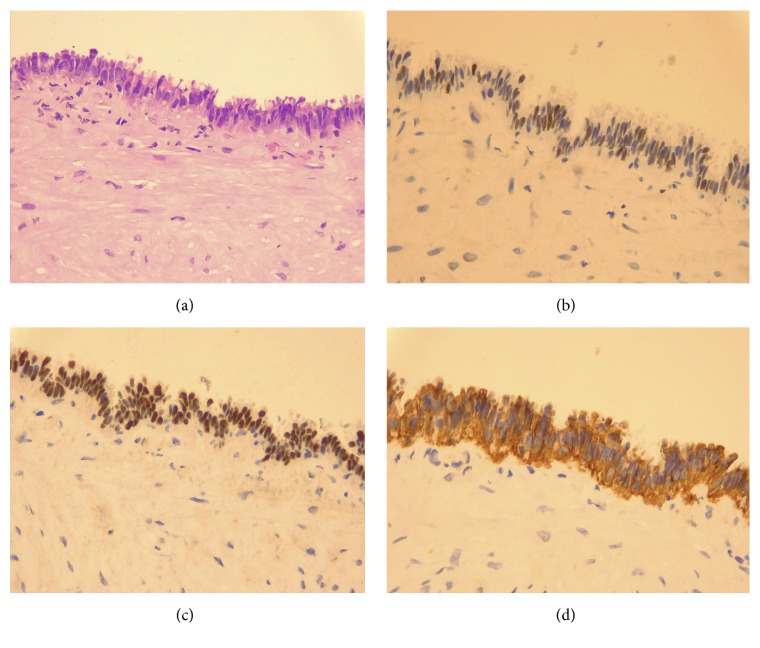
Microscopic view of the cystic endosalpingiosis lesions. The hematoxylin-eosin stain (a) showed a histology formed by an external serous layer, a well-organized smooth muscle and an inner layer of tubal cylindrical epithelium with small fibrous stroma papillae, no atypias, and absence of proliferative activity. Although some decidualized cells were present, no endometrial stroma was found. The immunohistochemistry analysis showed a positive expression for estrogen (b) and progesterone receptors, PAX8 (Box-8) (c) and CK7 (d), and a negative expression for CD10, calretinin, and CK20. The proliferative index with Ki67 was below 1%.

The results of the cysts biopsy (Figure 2) showed a histology formed by an external serous layer, a well-organized smooth muscle, and an inner layer of tubal cylindrical epithelium with small fibrous stroma papillae, no atypias, and no proliferative activity. Although some decidualized cells were present, no endometrial stroma was found. The immunohistochemistry analysis showed a positive expression for PAX8 (Box-8), CK7, and estrogen and progesterone receptors and a negative expression for CD10, calretinin, and CK20. The proliferative index with Ki67 was below 1%. The cytology showed histiocytes and scarce inflammatory cellularity. The final diagnosis was of florid cystic endosalpingiosis.

**Figure 3:**
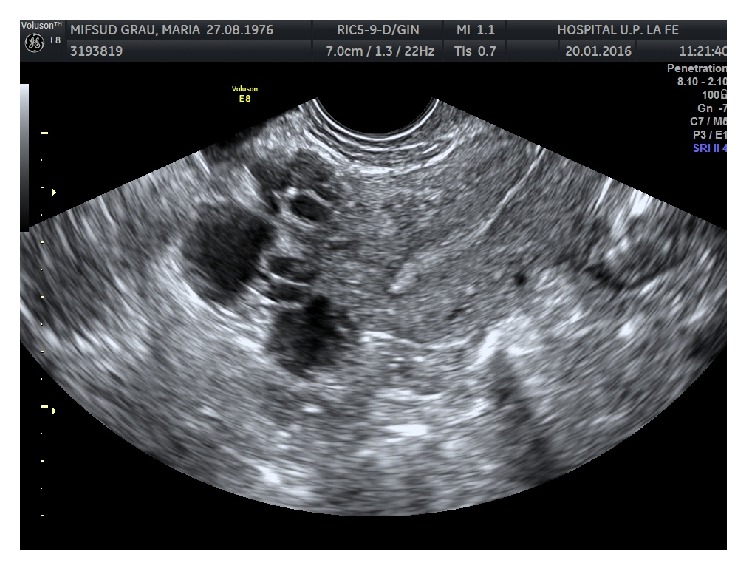
Transvaginal ultrasound image of the cystic endosalpingiosis lesions 3 months after the cesarean section. The fundus and part of the uterine body were still covered with multitude of cyst-like structures resembling hydatids of Morgagni. The endosalpingiosis lesions did not disappear after pregnancy.

Three months after the cesarean section, the patient was reevaluated with transvaginal ultrasound (Figure 3). The examination showed that the fundus and part of the uterine body were still covered with multitude of cyst-like structures. The endosalpingiosis lesions did not disappear after pregnancy.

## 3. Discussion 

Cystic endosalpingiosis is part of müllerianosis, disorders consisting in the heterotopic presence of müllerian-derived tissue [1, 2] in pelvic organs like the uterus [3], bladder [4], ovaries [5], parametrium [6], uterosacral mesosalpinx [7], peritoneum [8], and ureters [9] or in distant locations like the small [10] and large intestine (especially in the appendix) [11], coledochal duct [12], axillary nodes [13], mediastinum [14], umbilicus [15], vessels [16], and spine [17].

Most of the reported cases have been observed in nonpregnant women complaining of pelvic pain [18, 19] and urological [9], digestive [20], or neurological symptoms [21] after an ultrasound [22] or MRI [6] examination mimicking diverse kinds of pelvic cystic tumors [23]. Although müllerianosis may contain estrogen and progesterone receptors [14], reports of cystic endosalpingiosis and other forms of müllerianosis in pregnancy are surprisingly very scarce. They are considered choristomas (masses of normal tissue in an abnormal locations) causing endosalpingiosis, endometriosis, adenomyosis, endocervicosis, leiomyomatosis peritonealis disseminata, and probably vascular leiomyomatosis.

During organogenesis, a number of genes of the WNT family [24] like the WNT4 are activated, producing the necessary signals to conduct the development of the mullerian structures. That is the reason why mutations in the WTN-4 gene cause müllerian duct regression [25]. Recent research has underlined the possibility that, on the other extreme, müllerianosis might be caused by the abnormal reactivation of these genes [26, 27], causing metaplasia of normal tissues like the peritoneum. This would explain why these anomalies appear disseminated in the pelvic and abdominal organs [28, 29] or why Box-8 (PAX8) positive cells appear so frequently in peritoneal washing for diverse gynecological indications [30]. However it is true that another possibility for these findings would be the presence of remnants of müllerian precursor cells included within the developing tissues. Be that as it may, these cells are sensitive to estrogen and progesterone and might proliferate during pregnancy increasing the volume of cyst and thus making them detectable at the end of pregnancy. However, the fact that the lesions did not disappear after pregnancy makes this possibility less likely. In summary, cystic endosalpingiosis is a benign condition that should always be considered, even in pregnancy, when it comes to making the differential diagnosis of a pelvic or systemic multicystic mass.
